# Editorial: Adaptive immunity in atherosclerosis

**DOI:** 10.3389/fimmu.2024.1440283

**Published:** 2024-06-20

**Authors:** Bram Slütter, Klaus Ley

**Affiliations:** ^1^ Div. BioTherapeutics, Leiden Academic Centre for Drug Research, Leiden University, Leiden, Netherlands; ^2^ Immunology Center of Georgia, Medical College of Georgia at Augusta University, Augusta, GA, United States

**Keywords:** atherosclerosis, B-cell, T-cell, adaptive immunity, TCR sequencing

Atherosclerosis, the main underlying condition of acute cardiovascular events, has traditionally been regarded and treated as a disease caused by perturbed lipid homeostasis. Clinical trials in the last decade, however, have highlighted the critical role of chronic inflammation on disease outcomes. The adaptive immune system plays a critical role in propagating and regulating inflammation and may allow new, more specific, intervention strategies for atherosclerosis. The advent of new immunological techniques such as single-cell RNA sequencing, spectral flow cytometry, and mass cytometry has greatly expanded our insights into adaptive immune systems’ role in atherosclerosis development, progression and perhaps regression. With the subsequent detection of autoantibodies and autoreactive T-cells specific to cardiovascular patients, we now face the realization that atherosclerosis shares major commonalities with autoimmune diseases and could be treated as such. This greatly expands the therapeutic horizons for this disease.

Recent studies have shown that atherosclerosis is accompanied by a loss of tolerance (1, 2). This discovery has become possible through new technologies for paired TCRα and TCRβ sequencing using single-cell RNA-sequencing technology, thus defining clonotypes. This allows the determination of clonality, finding expanded clones, and understanding their phenotype. Modern tools to classify TCR sequences help make sense of the clonal expansion observed in atherosclerosis.

This Research Topic aims to summarize and extend the latest findings in adaptive immunity in atherosclerosis. It encompasses eight papers: five original reports and three reviews. The T cell papers report on single cell transcriptomes, T cell receptor (TCR) sequences and clonality, and T cell activation by the chemokine CCL18. Vos et al. found that T cell-specific deletion of CBL-B (CBL-B TKO) reduced atherosclerosis in the aortic arch and root, but modestly increased plaque T cell infiltration and systemic T cell activation. The number of CD8+ T cells in the spleen was elevated by 40% in CBL-B TKO mice compared to controls. Interestingly, the number of CD4+ regulatory T cells (Tregs) was elevated even more (by 80%). Tregs are known to be atheroprotective (3), suggesting that elevated numbers may be part of the mechanism that reduces atherosclerosis in CBL-B TKO mice. There were also markers of T cell activation and exhaustion. Iqneibi et al. used single cell transcriptomics of peripheral blood mononuclear cells (PBMCs) from 30 human subjects with coronary artery disease (CAD) and 30 subjects without CAD and found signs of recent CD8 TCR engagement. This shows that the previously described evidence for CD8+ T cell activation in the plaque (4) is also evident in PBMCs, which are much more accessible to clinical investigation than plaque. Interestingly, the gene most predictive of the presence of CAD was CTSW, a cytotoxic protease released from CD9+ T cells during cell killing. Roy et al. focused on CD4+ T cell responses to the known (5) atherosclerosis autoantigen apolipoprotein B (ApoB). They found CD4+ T cell responses to some of the HLA class-II restricted epitopes in 6 patients with different HLA alleles. Some ApoB-specific clones were highly expanded with up to 200 copies of the same TCRβ clonotype detected, showing the potential of these autoreactive T-cells to robustly respond to antigens. Before these T-cell can respond to antigen however, trafficking to the plaque is an essential mechanism, with interesting therapeutic option to intervene in the inflammatory cascade. Singh et al. studied the impact of CCL18 on T cell influx and polarization. They found CCL18 upregulated in ruptured human atherosclerotic plaques. In the ApoE knockout mouse model of atherosclerosis, they showed that CCL18 was able to induce skin inflammation, and this response was attenuated in CCR6-deficient mice, suggesting that CCR6 may be (one of the) receptor(s) for CCL18 and may be involved in driving T-cell influx into the unstable lesions.

Like T cells, B cells show enormous diversity. B1 cells are thought to be atheroprotective and most B2 cells are thought to be pro-atherogenic, however insights suggest their antigen specificity may determine how disease is impact. BCR sequencing has contributed to the identification autoreactive antibodies (6) and pro- and anti-atherogenic B cell subsets. In this topic Pattarabanjird et al. show the presence of a CD11c+ subset of B cells in patients with coronary artery disease. In mice, their frequency was positively correlated with plaque burden. In humans, more CD11c+ B cells were found in PBMCs from subjects with CAD. In humans and in the ApoE knockout mouse model of atherosclerosis, CD11c+ B cells increased with age. Thus, these cells are considered age-associated B cells (ABC). In line with these findings, Snijckers et al. explore the adaptive immune system in atherosclerosis during aging. One aspect of aging is immunosenescence, characterized by increase in central memory cells, a decrease in naïve T- and B cells, increased propensity for autoimmunity and a reduced the capacity for immune responses to new foreign antigens. They review the effects of age on T and B cells and how these changes might affect major adverse cardiovascular events (MACE). Adding to the topic aging and immunity.

Kral et al. provide a review focusing on innate lymphoid cells (ILCs), aging and efferocytosis in the context of atherosclerosis. They primarily reviewed ILC2, a cell type known to be atheroprotective. ILC2 cells are involved in tissue maintenance, repair, and type 2 inflammation. The atheroprotective effects seem to occur in plaque and perivascular adipose tissue, but the mechanisms remain unknown. A final review paper is provided by Ngai et al., who review the role of efferocytosis, the process by which macrophages take up apoptotic cells, in T cell activation. The authors lay out some hypotheses how efferocytosis might affect the adaptive immune system and may lead to T- and B-cell activation.

Although this Topic cannot cover all aspects of adaptive immunity in atherosclerosis, we think it provides a good overview. The three reviews are up-to-date primers suitable for new investigators who wish to work in these areas. The original articles are both interesting and timely. We hope you will enjoy reading this Topic.

## Author contributions

BS: Writing – original draft, Writing – review & editing. KL: Writing – original draft, Writing – review & editing.
